# Headspace Solid-Phase Microextraction Analysis of Volatile Components in *Phalaenopsis* Nobby’s Pacific Sunset

**DOI:** 10.3390/molecules190914080

**Published:** 2014-09-09

**Authors:** Chih-Hsin Yeh, Wan-Yu Tsai, Hsiu-Mei Chiang, Chin-Sheng Wu, Yung-I Lee, Li-Yun Lin, Hsin-Chun Chen

**Affiliations:** 1 Taoyuan District Agricultural Research and Extension Station, Council of Agriculture, Executive Yuan, Taoyuan 327, Taiwan; E-Mail: zeamays@tydais.gov.tw; 2 Department of Agronomy, National Taiwan University, Taipei 106, Taiwan; 3 Department of Cosmeceutics, China Medical University, Taichung 404, Taiwan; E-Mails: kh94j03m4@hotmail.com (W.-Y.T.); hmchiang@mail.cmu.edu.tw (H.-M.C.); cswu@mail.cmu.edu.tw (C.-S.W.); 4 Department of Biology, National Museum of Natural Science, Taichung 404, Taiwan; E-Mail: leeyungi@hotmail.com; 5 Department of Life Sciences, National Chung Hsing University, Taichung 402, Taiwan; 6 Department of Food Science and Technology, Hungkuang University, Taichung 433, Taiwan

**Keywords:** *Phalaenopsis*, volatile components, *Phalaenopsis* Nobby’s Pacific Sunset, headspace-solid phase microextraction (SPME)

## Abstract

*Phalaenopsis* is the most important economic crop in the Orchidaceae family. There are currently numerous beautiful and colorful *Phalaenopsis* flowers, but only a few species of *Phalaenopsis* have an aroma. This study reports the analysis volatile components present in *P.* Nobby’s Pacific Sunset by solid-phase microextraction (SPME) coupled with gas chromatography (GC) and gas chromatography/mass spectrometry (GC-MS). The results show that the optimal extraction conditions were obtained by using a DVB/CAR/PDMS fiber. A total of 31 compounds were identified, with the major compounds being geraniol, linalool and α-farnesene. *P.* Nobby’s Pacific Sunset had the highest odor concentration from 09:00 to 13:00 on the eighth day of storage. It was also found that in *P.* Nobby’s Pacific Sunset orchids the dorsal sepals and petals had the highest odor concentrations, whereas the column had the lowest.

## 1. Introduction

*Phalaenopsis*, a member of the Orchidaceae family and the *Phalaenopsis* genus, is of high economic and ornamental value. *Phalaenopsis* species, or hybrids thereof, are very popular ornamental flowers worldwide because of their beautiful appearance [1], and their flowering cycles, that can last up to one to three months. *Phalaenopsis* are mainly distributed in tropical and subtropical regions, including parts of southern China, the Philippines, India and northern Australia [2]. Recently, morphological polymorphism of *Phalaenopsis* has been found to commonly occur during the breeding of hybrids, especially in the floral organs, resulting in mostly scentless flowers that exhibit various colors, shapes and sizes [3]. However, there are currently several breeders striving to breed scented *Phalaenopsis* with enhanced economic value. 

There are only a few studies on the aroma of *Phalaenopsis*. Kaiser [4] reported the volatile constituents of *P.* *violacea* Borneo type and *P.* *violacea* Malaya type, of which the major components were linalool (49.0%) and geraniol (43.0%) for *P.* *violacea* Borneo type and linalool (27.8%) and elemicine (26.7%) for *P.* *violacea* Malaya type. Awano *et al.* [1] analyzed the volatile components of *P. schilleriana*, *P.* *equestris* and *P.* *veitchiana* by the headspace method, and the major components were determined to be neryl acetate (53.8%) for *P. schilleriana*, nonanal (13.7%) and decanal (19.9%) for *P.* *equestris* and citronellyl acetate (12.6%), decanal (13.0%) and 1,4-dimethoxybenzene (25.0%) for *P.* *veitchiana*. Hsiao *et al.* [5] identified 38 analyzed volatile compounds in *P.* *bellina* and *P.* *equestris*. The main components were linalool and *trans*-geraniol for *P.* *bellina* and 2,6-dimethoxy-4-(2-propenyl)phenol for *P.* *equestris.*

Headspace analysis can be used to determine the composition of living natural materials and to provide different and broader olfactory profiles [6]. SPME is a simple, fast, sensitive and convenient sample preparation technique that minimizes solvent usage while integrating sampling and sample preparation steps prior to instrumental analysis [7]. Recently, SPME has been widely applied to the sampling and analysis of environmental, food, aromatic, metallic, forensic, biological and pharmaceutical samples [8]. Several types of coating fibers are currently available for the extraction of analytes. For example, non-polar polydimethylsiloxane (PDMS) fiber are preferred for the extraction of non-polar analytes, such as many volatile flavor compounds. CAR–PDMS fibers exhibit better extraction efficiency than 100 mm PDMS fibers and similar fibers, but display inferior repeatability and their equilibration is more time-consuming [9]. Several types of coating fibers are currently available for the extraction of analytes. For example, the PA phase is suitable for more polar compounds such as phenols, whereas the PDMS/DVB fiber can absorb aromatic compounds [9,10].

*P*. Nobby’s Pacific Sunset is a hybrid species that can bloom all year and the flower has a long shelf life. It also has a strong odor, while most *Phalaenopsis* hybrid flowers any lack any scent*.* The volatile components of this species have not yet been reported, therefore, this study aimed to apply SPME to the analysis of the aroma of *P*. Nobby’s Pacific Sunset, determine the optimal SPME adsorptive fiber and extraction time and monitor the changes in odor during the flowering cycle over 24 h as well as various stages of the flowering period. The differences in aroma in different parts of the *Phalaenopsis* were also explored.

## 2. Results and Discussion

### 2.1. Comparison Optimal of SPME Fiber Coating

**Figure 1:**
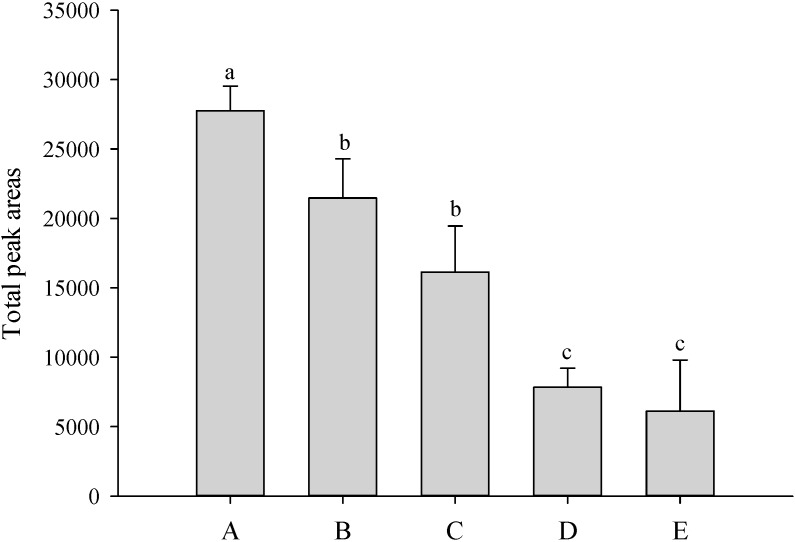
Comparison of the contents of total volatile compounds detected in headspace of *P*. Nobby’s Pacific Sunset by different SPME fibers (A: DVB/CAR/PDMS; B: CW/DVB; C: PDMS/DVB; D: polyacrylate; E: PDMS). Values are means ± SD of triplicates. Values with different superscripts are significantly (*p* < 0.05) different.

Five different SPME fibers (100 μm PDMS, 85 μm polyacrylate, 65 μm CW/DVB, 85 μm CAR/PDMS and 50/30 μm DVB/CAR/PDMS) were used to adsorb the volatile constituents of *P*. Nobby’s Pacific Sunset, respectively. The results determined that fibers with 50/30 μm DVB/CAR/PDMS had optimal extraction ability (Figure 1). 

SPME fiber coatings are available in a wide assortment of thicknesses and polarities [11]. Adam *et al.* [12] used different fibers (100 μm PDMS, 65 μm PDMS/DVB and 50/30 μm DVB/CAR/PDMS) to extract volatile compounds of yacon leaves. The best results for most of the target compounds were obtained using the DVB–CAR–PDMS fiber, which can be explained by the nature of the fiber, as well as its slightly larger capacity for the analytes. Stashenko *et al.* [11] reported that PDMS is a non-polar material such as DVB and CAR. Lee *et al.* [13] used SPME to analyze the volatile constituents of garlic flavor components and compared the extraction abilities of the five different adsorptive fibers. The DVB/CAR/PDMS fiber was found to be the most efficient among the five types of fibers investigated.

### 2.2. Analysis of the Volatiles in P. Nobby’s Pacific Sunset Flowers

The volatile compounds in *P.* Nobby’s Pacific Sunset flowers were analyzed by solid-phase microextraction coupled with GC and GC-MS. A total of 31 components were identified in *P*. Nobby’s Pacific Sunset, including 14 terpenes, five terpene alcohols, four aldehydes, three esters, two hydrocarbons, one ketone, and two other compounds (Table 1). The main constituents were geraniol (28.31% ± 8.97%), linalool (28.30% ± 16.78%) and α-farnesene (11.10% ± 4.49%). 

**Table 1:** Percentages of volatile compounds of *P.* Nobby’s Pacific Sunset.

RI ^a^	Compound	Content (%)
641	2-methylbutanal	0.60 ± 0.15
882	styrene	0.02 ± 0.00
933	benzaldehyde	0.08 ± 0.02
964	6-methyl-5-hepten-2-one	0.69 ± 0.55
981	β-myrcene	3.87 ± 0.18
1021	limonene	1.30 ± 0.04
1023	*cis*-β-ocimene	2.17 ± 0.29
1035	*trans*-β-ocimene	0.07 ± 0.02
1050	α-ocimene	0.10 ± 0.01
1060	3-methylphenol	0.07 ± 0.02
1065	2-methylphenol	0.22 ± 0.10
1080	linalool	28.30 ± 16.78
1099	(*E*)-4,8-dimethyl-1,3,7-nonatriene	0.82 ± 0.08
1189	methyl salicylate	0.07 ± 0.02
1201	nerol	4.54 ± 1.05
1231	geraniol	28.31 ± 8.97
1233	geranial	2.04 ± 0.17
1357	neryl acetate	0.91 ± 0.57
1360	geranyl acetate	0.52 ± 0.26
1444	*trans*-β-farnesene	1.08 ± 0.87
1469	α-humulene	0.17 ± 0.13
1477	α-curcumene	0.29 ± 0.19
1481	(*Z*,*E*)-α-farnesene	0.47 ± 0.07
1489	α-zingiberene	0.20 ± 0.10
1490	*cis*-γ-bisabolene	<0.01
1495	(*E*,*E*)-α-farnesene	11.10 ± 4.49
1544	nerolidol	0.31 ± 0.25
1556	*trans*-γ-bisabolene	0.17 ± 0.04
1686	*cis*-11-hexadecenal	0.10 ± 0.07
1705	(*Z*,*E*)-farnesol	1.18 ± 0.82
1721	(*E*,*E*)-farnesal	0.17 ± 0.15
	Total	89.91 ± 1.19

^a^ Retention indices, using paraffin (C_5_-C_25_) as references.

Among these main compounds, the odor of geraniol is rose-like and geranium-like as described by Rega *et al.* [14] and Eyres *et al.* [15]. Högnadóttir *et al.* [16] also indicated that linalool has a floral aroma, and is found in flowers such as *Michelia alba*, *Chimonanthus praecox*, *Momordica charantia*, Hop, *Origanum vulgare* and *Camellia sinensis*. Linalool is a naturally-occurring terpene alcohol found in many flowers to attract pollinators, but with anti-ethylene and anti-inflammatory functions [17,18,19,20,21,22,23]. α-Farnesene has a wood-like, floral and weak spicy aroma [24,25]. The odor of nerol is sweet floral as described by Zhao *et al.* [26]. Tamura [27] cited that myrcene has a warm grassland-like and green‑mango-like aroma, and at concentrations below 10 ppm, it has a sweet herbal aroma with citrus notes [28].

#### 2.2.1. Volatile Compounds of *P*. Nobby’s Pacific Sunset Flowers over a 24 h Period

**Figure 2:**
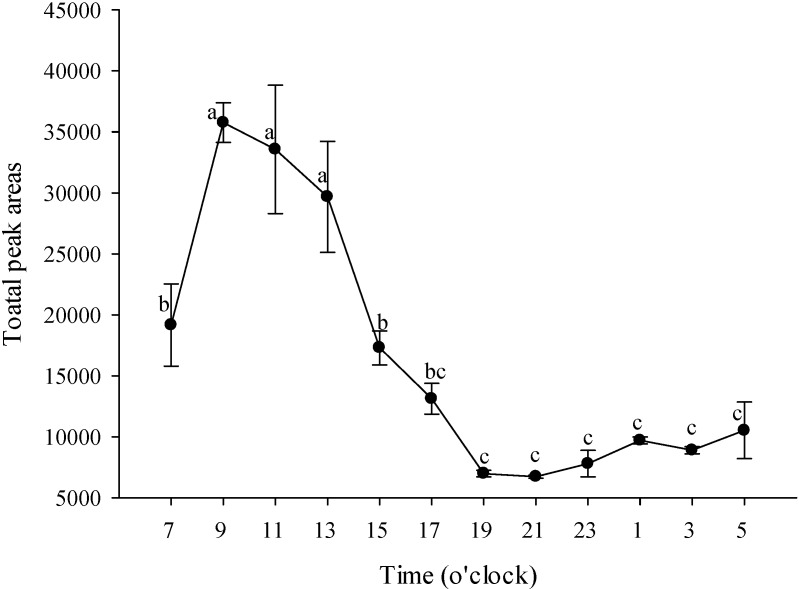
Changes in the volatile components of *P*. Nobby’s Pacific Sunset over a 24 h period. Values are means ± SD of triplicates. Values having different superscripts are significantly (*p* < 0.05) different.

Figure 2 shows that the odor was the strongest at 09:00 and gradually declined until 13:00, at which point it began to decline rapidly. Awano *et al.* [1] studied the differences in the concentrations of *P. schilleriana* at three stages during the day (05:00 to 13:00, 13:00 to 21:00 and 21:00 to 05:00) and found that during the first stage approximately 87% of the volatiles were emitted. They also reported that *P. schilleriana* began to emit the volatiles just after sunrise, reaching a peak of scent production 4–5 h later, and becoming nearly scentless after approximately 15:00.

**Figure 3:**
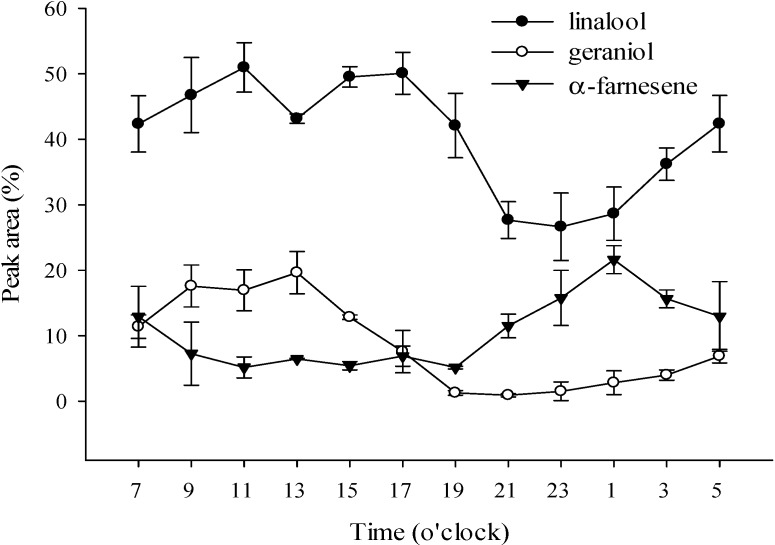
Changes in the major volatile components of *P*. Nobby’s Pacific Sunset over a 24 h period.

The odor of flowers changes at different times to attract day and night pollinators. The main constituents of *P*. Nobby’s Pacific Sunset, α-farnesene, geraniol and linalool, also change at different times (Figure 3). The results showed that linalool was the main constituent throughout the entire day, wheres α-farnesene increased and linalool decreased at night (19:00 to 03:00). Many studies have reported that linalool is known as an attractant for Apidae and Colletidae bees, but seems to be unattractive to Halictidae bees and brachyceran flies [29,30,31]. Christenson [2] noted that the pollinating insects of *Phalaenopsis* are bees, which are diurnal; therefore, it can be inferred that this is the reason linalool decreases at night. There is an advantage for the plant to have its scent output at maximal levels only when the flower is ready for pollination and concomitantly when its potential pollinator is active [32]. *C. breweri* flowers, despite being moth-pollinated, do not show noticeable differences in emissions between day and night. Snapdragon flowers, on the other hand, are bee-pollinated and show a marked peak of emission during the day [33].

#### 2.2.2. Volatile Compounds of *P.* Nobby’s Pacific Sunset Flowers in a Blooming Cycle

**Figure 4:**
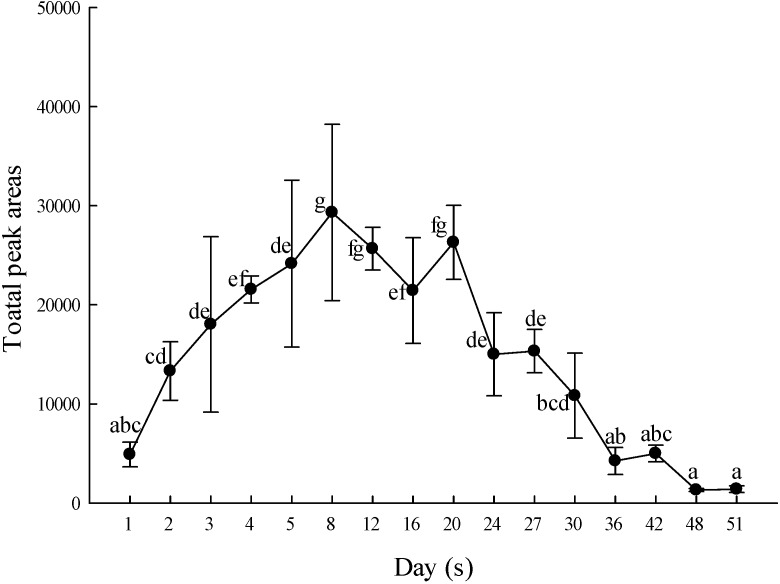
Changes in the volatile components of *P*. Nobby’s Pacific Sunset during the blooming cycle. Values are means ± SD of triplicates. Values having different superscripts are significantly (*p* < 0.05) different.

**Figure 5:**
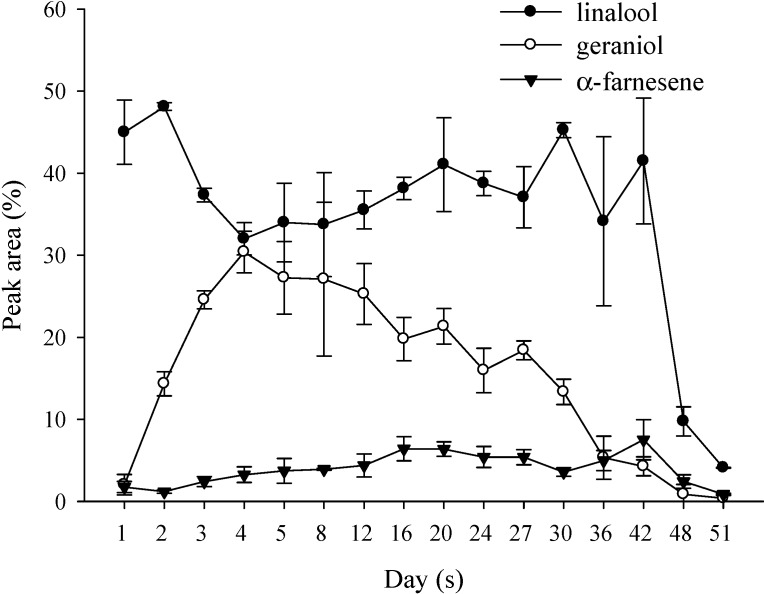
Changes in the major volatile components of *P*. Nobby’s Pacific Sunset during the blooming cycle.

As shown in Figure 4, the volatile components content peaked on day 8, slowly decreasing thereafter. The ratios of the main compounds α-farnesene, geraniol and linalool also changed on different days. Linalool levels were reduced to 30% whereas geraniol increased to 30%. Therefore, it can be obviously found that the aroma of *P.* Nobby’s Pacific Sunset will change as a result of the flowering days (Figure 5). Hsiao *et al.* [34] reported that the highest aroma of *P. bellina* occurred during days 5–7. Zhang *et al.* [35] also indicated that for *Oncidium* Sharry Baby, during flower blooming and senescence, the aroma contents of alkenes, alcohols and esters increased, whereas the contents of aldehydes, ketones and alkanes decreased. Rusanov *et al.* [36] sought to determine the optimal timing for harvesting *Rosa damascena* flowers. Thus, the flowers were divided into eight different developmental stages for analysis. The results showed that the best harvesting time is during full bloom when the aroma is at its highest concentration but does not contain the carcinogenic component, methyl eugenol. Chen *et al.* [37] reported the vase-life of *Narcissus tazetta* var. *chinensis* Roem, the total volatile component content of which peaked on day 2 for single-flowered and day 3 for double-flowered narcissus, with both decreasing significantly by day 4.

### 2.3. Volatile Compounds of Different Parts of the P. Nobby’s Pacific Sunset Flowers

In this study, *P.* Nobby’s Pacific Sunset flowers were separated into five parts: petal, dorsal sepal, lateral sepal, lip and column. This study used SPME to extract the odor of each part of the *P*. Nobby’s Pacific Sunset flower and identified 80.98%–88.34% of its constituents (Table 2). 

**Table 2:** Percentages of volatile compounds in different parts of *P.* Nobby’s Pacific Sunset.

RI ^a^	Compound	Whole Flowers	Petals	Lateral Sepals	Dorsal Sepals	Lip	Column
641	2-methyl butanal	0.60 ± 0.15	0.26 ± 0.24	- ^b^	-	0.17 ± 0.12	-
882	styrene	0.02 ± 0.00	1.28 ± 1.16	0.86 ± 0.62	2.80 ± 1.78	-	-
933	benzaldehyde	0.08 ± 0.02	-	0.07 ± 0.00	0.07 ± 0.00	0.05 ± 0.02	-
964	6-methyl-5-hepten-2-one	0.69 ± 0.55	-	0.48 ± 0.24	-	-	-
981	β-myrcene	3.87 ± 0.18	6.83 ± 0.05	6.35 ± 0.46	5.93 ± 0.45	5.62 ± 1.33	4.89 ± 0.21
1021	limonene	1.30 ± 0.04	2.27 ± 0.02	2.12 ± 0.34	-	1.95 ± 0.39	-
1023	*cis*-β-ocimene	<0.01	-	-	-	-	2.39 ± 0.32
1035	*trans*-β-ocimene	2.16 ± 0.28	3.60 ± 1.03	2.98 ± 0.22	3.21 ± 0.65	2.53 ± 0.68	6.44 ± 1.62
1050	α-ocimene	0.07 ± 0.02	0.10 ± 0.01	0.10 ± 0.03	0.08 ± 0.02	0.12 ± 0.01	0.35 ± 0.02
1060	3-methylphenol	0.07 ± 0.02	0.10 ± 0.01	-	-	-	-
1065	2-methylphenol	0.22 ± 0.10	0.33 ± 0.02	0.32 ± 0.13	0.29 ± 0.06	0.53 ± 0.06	0.84 ± 0.24
1080	linalool	28.30 ± 16.78	40.24 ± 2.77	39.53 ± 7.40	42.04 ± 3.08	67.77 ± 4.98	63.53 ± 6.57
1099	(*E*)-4,8-dimethyl-1,3,7-nonatriene	0.51 ± 0.06	1.33 ± 0.15	1.20 ± 0.13	1.90 ± 0.29	0.80 ± 0.05	-
1189	methyl salicylate	0.11 ± 0.05	-	-	-	-	-
1201	nerol	4.54 ± 1.05	2.40 ± 0.77	-	-	-	-
1231	geraniol	28.31 ± 8.97	15.68 ± 4.39	17.77 ± 6.43	9.88 ± 2.07	6.50 ± 1.92	-
1233	geranial	-	1.15 ± 0.12	1.17 ± 0.15	-	-	-
1357	neryl acetate	0.91 ± 0.57	-	-	-	0.59 ± 0.53	-
1360	geranyl acetate	0.52 ± 0.26	-	-	-	-	-
1444	*trans*-β-farnesene	1.08 ± 0.87	-	-	-	-	-
1469	α-humulene	0.27 ± 0.07	-	-	-	-	-
1477	α-curcumene	0.29 ± 0.19	0.48 ± 0.16	0.46 ± 0.08	0.57 ± 0.11	0.16 ± 0.03	-
1481	(*Z*,*E*)-α-farnesene	0.47 ± 0.07	-	-	-	-	-
1489	α-zingiberene	0.20 ± 0.10	-	-	-	-	-
1490	*cis*-γ-bisabolene	<0.01	-	-	-	-	-
1495	(*E*,*E*)-α-farnesene	11.10 ± 4.49	10.33 ± 1.27	10.71 ± 3.10	13.73 ± 1.32	2.59 ± 0.47	4.83 ± 3.08
1544	nerolidol	0.31 ± 0.24	-	-	-	-	-
1556	*tran*s-γ-bisabolene	0.17 ± 0.05	-	-	-	-	-
1686	*cis*-11-hexadecenal	0.19 ± 0.05	-	0.03 ± 0.03	-	-	-
1705	(*Z*,*E*)-farnesol	0.82 ± 0.85	-	-	-	-	-
1721	(*E*,*E*)-farnesal	0.17 ± 0.15	-	-	-	-	-

^a^ Retention indices, using paraffin (C_5_-C_25_) as references; ^b^ undectable.

**Figure 6:**
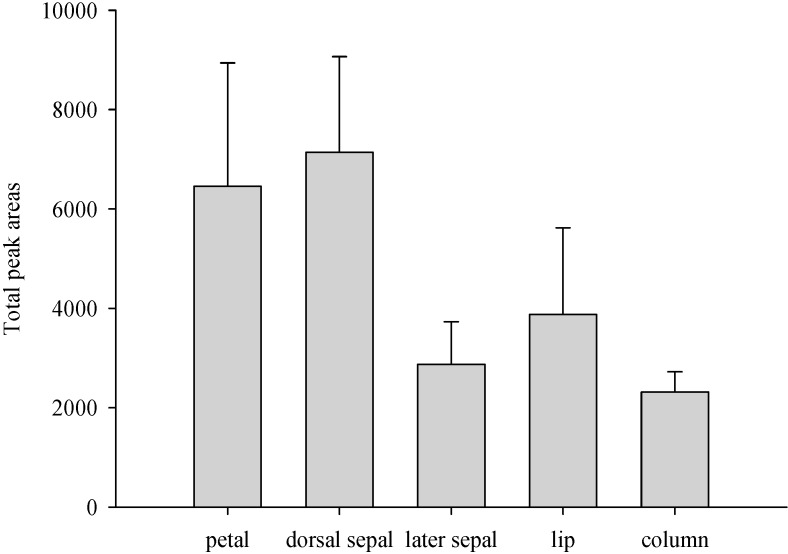
The relative concentration of floral scent from different parts of the *P*. Nobby’s Pacific Sunset flower.

As shown in Figure 6, the part with the strongest odor was the dorsal sepal and the weakest was found in the column. The major compound found in the above-mentioned flower parts was linalool. The secondary volatile constituents of the petal and dorsal sepal were geraniol and α-farnesene. The content of α-farnesene was higher than that of geraniol in the lateral sepal. Geraniol and β-myrcene were the secondary volatile constituents of the lip, and *trans*-β-ocimene and β-myrcene were the main volatile constituents of the column.

In addition, the petal and dorsal sepal of *Phalaenopsis* had similar aroma composition ratios, as did the lip and column, except that the column did not contain geraniol. Kilic *et al.* [38] reported that the major volatile components of fresh leaves, buds, flowers and fruits from bay (*Laurus nolilis* L.) was linalool, which gives a floral aroma, as determined by gas chromatography-olfactometry-mass spectrometry (GC-O). Linalool is a floral aromatic compound that attracts pollinators, inhibits ethylene action and induces processes, and it has demonstrated anti-inflammatory activities [21].

The results showed that each part of the flower contains terpenes, such as *trans*-β-ocimene, β-myrcene and α-farnesene, among which α-farnesene content in the lip and column differed the most from the α-farnesene content found in other parts. In addition, among the five parts, the column had the lowest aroma, but was also the only part that contained *cis*-β-ocimene (2.39% ± 0.32%). Among the terpene constituents, linalool exists in each part (39.53%–67.77%), with the lip containing the highest amount. Geraniol was not found in the column and had the highest concentration in the lateral sepal. 

Dobson *et al.* [39] analyzed volatiles from whole flowers, petals, sepals and sepals as well as gynoecium, anthers and pollen from *Rosa rugosa*. The results showed that petal volatiles, dominated by terpenoid and benzenoid alcohols, contributed most to the whole-flower fragrance. Sepal odors contained mainly sesquiterpenes. Compounds typical of the androecium were present as significant components (though in small amounts) of the whole-flower fragrance, which may function as signals to pollen-seeking insects.

## 3. Experimental Section

### 3.1. Plant Materials

*P*. Nobby’s Pacific Sunset flowers were collected in a greenhouse controlled at 25 °C located at the National Museum of Natural Science in Taichung, Taiwan.

### 3.2. Methods

#### 3.2.1. Comparison of SPME Fiber Coating

Five different coated SPME fibers, 100 μm polydimethylsiloxane (PDMS), 85 μm polyacrylate (PA), 65 μm carboxen/polydimethylsiloxane (CAR/PDMS), 85 μm carboxen/divinybenzene (CAR/DVB) and 50/30 μm divinybenzene/carboxen/polydimethylsiloxane (DVB/CAR/PDMS) fiber (Supelco, Inc., Bellefonte, PA, USA) were used for the aroma extraction. Fresh *P*. Nobby’s Pacific Sunset flowers in full bloom (ten each) were placed into a gas-collecting bag (1050-TK-3 MT passive bag; GL Sciences Inc., Tokyo, Japan). The SPME method was used to extract the aroma components. This experiment and all other experiments in this study were performed in triplicates.

#### 3.2.2. Volatile Components of *P*. Nobby’s Pacific Sunset

(1) Analysis of the changes in the volatile components of *P*. Nobby’s Pacific Sunset during a blooming cycle over a 24 h period: Changes in volatile components of *P*. Nobby’s Pacific Sunset (one flower each) were obtained over a 24 h period by covering each flower with a gas collecting bag at 01:00, 03:00, 05:00, 07:00, 09:00, 11:00, 13:00, 15:00, 17:00, 19:00, 21:00 and 23:00. The SPME method was used to adsorb and collect the components to determine changes in their amount over a day cycle.

(2) Volatile compounds during a blooming cycle of *P*. Nobby’s Pacific Sunset: Fresh budding *P*. Nobby’s Pacific Sunset flowers (one flower each) were selected on a given day that was defined as day 0. From day 0 to day 51 and from 09: 00–13: 00, the SPME method was used to extract and monitor the change in the aroma. 

(3) Isolation and analysis of the volatile compounds in different parts of the flowers: Ten fresh flowers were picked and separated into five parts: petal, dorsal sepal, lateral sepal, lip and column. The five parts were separately and immediately placed into sealed bottles (precleaned # 27343 22-mL clear screw cap vials; Supelco Inc.). The SPME method was used for adsorption and collection to determine the volatile compounds present.

#### 3.2.3. Analysis of Volatile Compounds

(1) HS-SPME analysis: A 50/30-μm divinylbenzene/carboxen/polydimethylsiloxane fiber (Supelco, Inc.) was used for aroma extraction. The SPME fiber was exposed to each sample for 60 min at 25 °C, after which each sample was injected into a gas chromatograph injection unit. 

(2) Analysis of the components of samples by GC and GC/MS: Qualitative and quantitative analyses of the volatile compounds were conducted using an Agilent 6890 GC (Agilent Tenologies, CA, USA) equipped with a 60 m × 0.25 mm i.d. DB-1 fused-silica capillary column with a film thickness of 0.25 μm and a flame ionization detector. The injector and detector temperatures were maintained at 250 °C and 300 °C, respectively. The oven temperature was held at 40 °C for 1 min and then raised to 200 °C at 2 °C/min and held for 9 min. The carrier gas (nitrogen) flow rate was 1 mL/min. Kovats indices were calculated for the separated components relative to a C_5_-C_25_ *n*-alkanes mixture [40]. Percentage composition was calculated using the peak area normalization measurements.

(3) Analysis of the components of the samples by GC-MS: The volatile compounds were identified using an Agilent 6890 GC equipped with a 60 m × 0.25 mm i.d. DB-1 fused-silica capillary column with a film thickness of 0.25 μm coupled to an Agilent model 5973 N MSD mass spectrometer (MS). The injector temperature was maintained at 250 °C. The GC conditions in the GC-MS analysis were the same as in the GC analysis. The carrier gas (helium) flow rate was 1 mL/min. The electron energy was 70 eV at 230 °C. The constituents were identified by matching their spectra with those recorded in a MS library (Wiley 7n). In addition, the constituents were confirmed by comparing the Kovats indices or GC retention time data with those of authentic standards or published in the literature. 

(4) Statistical Analysis: The data were subjected to a mono-factorial variance analysis with Duncan’s multiple range method utilized with a significance of differences of *p* < 0.05 (SPSS Base 12.0). 

## 4. Conclusions

Thirty-one volatile compounds were identified in *P.* Nobby’s Pacific Sunset. The main components were geraniol, linalool and α-farnesene. The fibers with 50/30 μm DVB/CAR/PDMS had the optimal extraction ability. The highest concentration of aroma of *P*. Nobby’s Pacific Sunset flowers occurred during 09:00 and 13:00, decreasing significantly by 15:00. During the blooming cycle, it was found that the total volatile content peak area was the greatest for *P.* Nobby’s Pacific Sunset flower on day 8, slowly decreasing thereafter. In addition, five parts of the *Phalaenopsis* flower were found to have similar characteristics, with linalool representing the primary compound. The dorsal sepal and petal were found to have the highest aroma, whereas the column had the lowest aroma.
